# Factors influencing the prevalence of cervical cancer screening in Malaysia: a nationwide survey

**DOI:** 10.1186/s12905-023-02553-3

**Published:** 2023-07-25

**Authors:** Yee Mang Chan, Muhd Zulfadli Hafiz Ismail, Wan-Fei Khaw

**Affiliations:** 1 Institute for Public Health, National Institutes of Health, Ministry of Health, Shah Alam, 40170 Malaysia; 2 Sector for Biostatistics and Data Repository, National Institutes of Health, Ministry of Health, Shah Alam, 40170 Malaysia

**Keywords:** Cervical cancer, Pap smear, Screening, Physical activity, Malaysia

## Abstract

**Background:**

In 2020, cervical cancer ranked fourth in terms of both frequency of diagnosis and the leading cause of cancer-related deaths among women globally. Among Malaysian women, it was the third most prevalent form of cancer. Published data on nationally representative cervical cancer screening in Malaysia have been limited. Therefore, this study aimed to determine the prevalence of receiving a Pap smear test in the past three years, its relationship with socio-demographic factors and physical activity.

**Methods:**

Using a subset of survey data from the National Health and Morbidity Survey (NHMS) 2019, a secondary data analysis was performed. Trained research assistants collected data through face-to-face method using a mobile tablet questionnaire system application. Logistic regression analysis was performed to examine the relationship between sociodemographic factors, physical activity, and cervical cancer screening. The analyses were conducted using STATA version 14 (Stata Corp, College Station, Texas, USA), accounting for sample weighs and complex sampling design.

**Results:**

The analysis included 5,650 female respondents, representing an estimated 10.3 million Malaysian female adults aged 18 and above. Overall, 35.2% (95%CI 33.2, 37.4) respondents had a Pap smear test within the past three years. Respondents who were physically active were 1.41 times more likely to have a Pap smear test. Similarly, respondents aged 35–59 (OR 1.84; 95%CI 1.46, 2.34) and those living in rural localities (OR 1.38; 95%CI 1.13, 1.70) had higher odds of receiving a Pap smear test. Compared to married respondents, single respondents (OR 0.04; 95%CI 0.02, 0.07) and widowed/divorcee respondents (OR 0.72; 95%CI 0.56, 0.82) were less likely to receive a Pap smear test. Educated respondents were more likely to have had a Pap smear test.

**Conclusions:**

The overall prevalence of cervical cancer screening in Malaysia remains low (35.2%). Efforts should be made to strengthen health promotion programs and policies in increasing awareness on the significance of cervical cancer screening. These initiatives should specifically target younger women, single women, and widowed/divorced individuals. The higher cervical screening uptake among rural women should be studied further, and the enabling factors in the rural setup should be emulated in urban areas whenever possible.

## Background

Cervical cancer is one of the most commonly diagnosed cancers in women. It was the fourth most frequently diagnosed cancer and the fourth leading cause of cancer death among women worldwide in 2020 [1]. During the same year, the estimated number of cervical cancer cases and deaths globally was 604,127 cases and 341,831 deaths respectively, with a corresponding age-standardised incidence of 13.3 cases per 100,000 women-years (95% CI 13.3–13.3) and a  mortality rate of 7.2 deaths per 100,000 women-years (95% CI 7.2–7.3) [2].

Low- and middle-income countries account for 84% of new cervical cancer cases and 87 − 90% of cervical cancer deaths [3]. Furthermore, countries with a low Human Development Index had three times higher incidence of cervical cancer compared to countries with a very high Human Development Index [2]. Despite cervical cancer being potentially preventable, it is the third most prevalent cancer among Malaysian women [4]. The HPV information centre estimates 1740 women were diagnosed with cervical cancer in Malaysia in 2020, with 991 succumbing from the disease [5].

Cervical cancer screening is one of the most cost-effective methods for early detection of the disease, and cervical cancer death is preventable. The 73rd World Health Assembly (August 2020) passed the resolution on a global strategy for cervical cancer elimination. This WHO global strategy calls for (i) 90% of girls to be fully vaccinated with the HPV vaccine by the age of 15, (ii) 70% of women to be screened using a high-performance test by the age of 35, and again by the age of 45, and (iii) 90% of women with pre-cancer to be treated and 90% of women with invasive cancer to be managed [6]. Despite past efforts, the self-reported lifetime prevalence of cervical cancer screening in 55 low- and middle-income countries was 44% from 2005 to 2018 [7]. According to a review and synthetic analysis, it was estimated that 133 million (84%) of 158 million women aged 30–49 years living in high-income countries had been screened for cervical cancer in their lifetime, compared to 194 million (48%) of 404 million women in upper-middle-income countries, 34 million (9%) of 397 million women in lower-middle-income countries, and 8 million (11%) of 74 million in low-income countries [8].

A study conducted in Malaysia’s northern state found that 38.6% women had undergone a Pap smear test in the past five years [9]. In contrast, a study among female staff in a tertiary hospital revealed that 33.5% had undergone a Pap smear test in the past three years [10]. According to a cross-sectional study conducted in the Malaysia’s southern state, 48.5% women visiting outpatient clinics had undergone Pap smear screening in the past three years [11]. The prevalence of having a Pap smear test done once in their lifetime ranges from 55.2% to 55.7% in Malaysia [9, 10]. The rate of cervical cancer screening is similar to that of surrounding countries. In China, lifetime cervical cancer screening coverage reached 43.4% in 2018–2019 [12]. In 2007 and 2009, the rates of cervical cancer screening coverage in Thailand were 46.3% and 59.7%, respectively [13].

A nationally representative cross-sectional study of non-institutionalized women in Spain reported that women who were older, had received a higher level of education and were from a higher social class were more likely to have cytology testing for cervical cancer screening [14]. Among Ethiopian women, determinant factors of cervical cancer screening uptake include knowledge of cervical cancer and screening, history of multiple sexual partners, women’s age, history of sexually transmitted disease, perceived susceptibility to cervical cancer, getting advice from health care providers, women’s educational level, and women’s attitude towards cervical cancer and screening [15]. American Indian women who are more physically active have higher rates of Papanicolaou test (pap smear) testing [16].

Human papillomavirus (HPV) is responsible for nearly all cases of cervical cancer [17]. The presence of persistent high-risk HPV infection may give rise to the formation of precancerous or invasive cancerous lesions. Cervical cancer cases are primarily caused by persistent genital high-risk HPV infection, accounting for approximately 99.7% of the cases [18]. Radiotherapy and surgery are equally effective in achieving positive oncological outcomes for early-stage cervical cancer, hence they serve as comparable treatment choices [19]. Research findings indicate that among women diagnosed with high-grade cervical dysplasia who undergo cervical conization, approximately 5% are classified as high-risk individuals due to the combination of positive cervical margins and persistent high-risk HPV infection [20]. Moreover, available evidence suggests that the safety of the minimally invasive approach to radical hysterectomy is still a concern [21]. According to a retrospective multi-institutional study, which compared minimally invasive and open radical hysterectomy in low-risk early-stage cervical cancer patients, laparoscopic radical hysterectomy does not lead to inferior 10-year outcomes compared to the open approach [22].

In Malaysia, there has been a scarcity of nationally representative published data on cervical cancer screening. To date, no study has been conducted in Malaysia to investigate the relationship between physical activity and cervical cancer screening. Therefore, this study aimed to determine the prevalence of receiving a Pap smear test in the past three years, as well as its relationship with socio-demographic factors and physical activity.

## Methods and materials

### Study design and sampling

The data for this study were derived from a subset survey of female adults aged 18 and above in the 2019 National Health and Morbidity Survey (NHMS).

### The national health and morbidity survey 2019 (NHMS 2019)

The NHMS 2019 is a nationwide community-based health survey conducted in Malaysia. This survey was carried out across all 13 Malaysian states and three federal territories, including both urban and rural areas. To achieve national representativeness, a two-stage stratified sampling method was adopted. Contiguous geographic areas, known as Enumeration Blocks (EBs), were derived from the entire Malaysia with the assistance of the Department of Statistics Malaysia (DOSM). These EBs served as the sampling frame. The EB was the first stage sampling unit, and the Living Quarters was the second stage sampling unit (LQs).

A total of 5,676 LQs were drawn from the 475 EBs selected across Malaysia, with 362 from urban areas and 113 from rural areas. Each EB had twelve LQs chosen at random. The study included all households within the selected LQs, as well as all household members. All individuals who had their primary residence and had lived in the selected LQ for at least two weeks prior to data collection were eligible to participate in this survey. Out of 5,676 LQs, 5,147 were deemed eligible for the study.

Out of the 5,147 eligible LQs, a total of 4,703 LQs were successfully interviewed, yielding a LQ response rate of 91.4%. These LQs qualified a total of 15,683 people for interviews. A total of 14,965 respondents were successfully interviewed, resulting in 95.4% individual response rate. As a result, the overall response rate for this community-based survey was 87.2%. NHMS 2019’s detailed methodology is described elsewhere [23].

### Data collection

Prior to data collection, a structured bilingual (Malay and English) questionnaire was designed, pre-tested, and piloted. Face-to-face interviews were used to collect data from July to October 2019. Data was collected by trained research assistants using a mobile tablet questionnaire system application.

The study was conducted in accordance with the principles of the Helsinki Declaration. The Malaysian Ministry of Health’s Medical Research and Ethics Committee approved the study, and it was registered in the National Medical Research Registry under the registration number NMRR-18-3085-44207. Prior to data collection, we obtained informed consent from each respondent.

### Study variables

#### Dependent variable

Respondents were asked: “In the past three years, did you do Pap smear examination?”. The possible responses were yes or no.

#### Independent variables

Sociodemographic factors included age group, locality, ethnicity, marital status, education level and employment. Age group was categorised into three groups: 18–34, 35–59 and, age 60 and above. Locality had two categories: urban and rural. Ethnicity was grouped into Malay, Chinese, Indian, Other Bumiputera and Others. Marital status was divided into three categories: married, single and widowed/divorcee. Education level had four categories: no formal education, primary education, secondary education and tertiary education. Employment status was grouped into yes and no. Physical activity was assessed using the validated short version of the International Physical Activity Questionnaire (IPAQ), with responses categorized into active and inactive.

### Data analysis

Weighting was used to adjust for the complex study design. To account for non-response and the varied probability of selection, each respondent was assigned a weighting factor. The estimation weight was determined by:

$${\rm{W = W1 \times W2 \times F \times PS}}$$

W1 = the inverse of probability of selecting the EBs, W2 = the inverse of probability of selecting the LQs within the EBs, F = the inverse of an EBs, LQs and individual level non-response adjustment factor, PS = a post stratification adjustment factor calculated by strata and gender.

The analysis only included respondents who had complete data on all variables required for this study, as the percentage of missing data was less than 5% [24]. Descriptive analysis was performed to describe the sociodemographic characteristics of respondents as well as the characteristics of study participants who had undergone a Pap smear test in the past three years.

The logistic regression analysis was performed to determine the association between independent variables and receiving a Pap smear test. Firstly, univariable associations between independent variables and receiving a Pap smear test were tested. Crude odds ratios (OR) were used to estimate the strength of association between independent and dependent variables. Following that, we included independent variables with p-value less than 0.25 in the multivariable regression models (25). The Variance Inflation Factor (VIF) test was used to examine the multicollinearity of independent variables in the final model. The VIF cut-off used in this study was 5 [25]. We examined the goodness of fit of the final model using Hosmer-Lemeshow goodness of fit test, classification table and ROC (receiver operating characteristic) curve.

All analyses were performed using STATA version 14 (Stata Corp, College Station, Texas, USA), taking into consideration the sample weighs and complex sampling design.

## Results

The analysis included 5,650 female respondents, representing an estimated 10.3 million Malaysian female adults aged 18 and above. Table 1 depicts the sociodemographic characteristics of the study respondents. The majority of the study respondents were Malay (52.8%), married (65.9%) and received secondary level education (47.2%). Slightly more than half of the study respondents reported being unemployed (53.4%). More than two-thirds of the study respondents were from urban locality (78.3%) and physically active (72.2%). Table 2 displays the characteristics of study respondents who undergone a Pap smear test in the past three years. Overall, 35.2% (95% CI 33.2, 37.4) of respondents had a Pap smear in the past three years. With the exception of age and marital status, those who had a Pap smear in the past three years shared similar characteristics.

Table 3 shows the results of univariable and multivariable logistic regression for Pap smear test uptake among adults aged 18 years and above. The variables age group, ethnicity, marital status, education level and physical activity had significant associations with Pap smear test uptake in the univariable analysis. The multivariable model included all variables except employment status.

After controlling for other variables, the multivariable model revealed that physically active respondents were more likely to have undergone a Pap smear test in the past three years (OR 1.41; 95%CI 1.12, 1.78). Respondents aged 35–59 had higher odds of receiving a Pap smear test in the past three years (OR 1.84; 95%CI 1.46, 2.34). Those living in rural areas were more likely to have undergone a Pap smear test in the past three years (OR 1.38; 95%CI 1.13, 1.70). When compared to married respondents, single respondents were less likely to have a Pap smear test in the past three years (OR 0.04; 95%CI 0.02, 0.07). Similarly, widowed/divorcee respondents were less likely to have had a Pap smear test in the past three years (OR 0.72; 95%CI 0.56, 0.82). Respondents with primary, secondary or tertiary education were more likely to have had a Pap smear test in the past three years.

In the multivariable model, the VIF test revealed no multicollinearity of independent variables (VIFs less than 5). We tested the goodness of fit of the multivariable model. The Hosmer-Lemeshow test revealed no evidence of poor fit (p = 0.332). Additionally, 67.13% of the observed values for the dependent outcome and the predicted values were correctly classified, suggesting that the assumption of classification table was met. The AUC of the ROC curve was 0.74, indicating that the model can precisely differentiate 74% of the cases. In summary, the multivariable model demonstrated a good fit.

**Table 1:** Sociodemographic characteristics of study respondents (N = 5,650)

Characteristics	Count, n (unweighted)	Estimated population, N (Weighted)	Percentage (%)
Pap smear			
No Yes	3359 2291	6,654,084 3,622,031	64.8 35.2
Age group (years)			
18–34 35–59 ≥60	1699 2715 1236	4,418,001 4,362,985 1,495,128	43.0 42.5 14.5
Locality			
Urban Rural	3435 2215	8,049,456 2,226,658	78.3 21.7
Ethnicity			
Malay^#^ Chinese Indian Other Bumiputera* Others	3671 701 362 615 301	5,425,938 2,196,477 641,046 1,168,746 843,906	52.8 21.4 6.2 11.4 8.2
Marital status			
Married Single Widowed/Divorcee	3765 967 918	6,770,641 2,326,903 1,178,570	65.9 22.6 11.5
Education level			
No formal education Primary Secondary Tertiary	478 1296 2535 1341	698,556 1,850,499 4,851,236 2,875,824	6.8 18.0 47.2 28.0
Employment status			
No Yes	3260 2390	5,487,790 4,788,324	53.4 46.6
Physical activity			
Inactive Active	1556 4094	2,861,834 7,414,281	27.8 72.2

^#^Includes indigenous people

*Includes Bumiputera Sabah and Sarawak

**Table 2:** Characteristics of respondents who undergone a Pap smear test in the past 3 years, (n = 2,291)

Characteristics	Count, n (unweighted)	Estimated population, N (Weighted)	Percentage (%)
Age group (years)			
18–34 35–59 ≥60	414 1471 406	954,019 2,217,218 450,794	26.3 61.2 12.4
Locality			
Urban Rural	1328 963	2,767,487 854,543	76.4 23.6
Ethnicity			
Malay^#^ Chinese Indian Other Bumiputera* Others	1508 271 169 261 82	1,967,134 798,463 243,909 431,023 181,501	54.3 22.0 6.7 11.9 5.0
Marital status			
Married Single Widowed/Divorcee	1926 29 226	3,169,704 67,106 385,221	87.5 1.9 10.6
Education level			
No formal education Primary Secondary Tertiary	116 528 1159 488	128,968 690,340 1,914,771 887,951	3.6 19.1 52.9 24.5
Employment status			
No Yes	1315 976	1,956,139 1,665,891	54.0 46.0
Physical activity			
Inactive Active	500 1791	734,096 2,887,935	20.3 79.7

^#^Includes indigenous people

*Includes Bumiputera Sabah and Sarawak

**Table 3:** Factors associated with cervical cancer screening among adults aged 18 years and above

Variables	Univariable model		Multivariable model	
	OR (95%CI)	p-value	aOR (95%CI)	p-value
Age group (years)				
18–34	*Ref*		*Ref*	
35–59	3.75 (3.03, 4.64)	< 0.001*	1.84 (1.46, 2.34)	< 0.001*
≥60	1.57 (1.21, 2.03)	< 0.001*	0.98 (0.72, 1.34)	0.882
Locality				
Urban	*Ref*		*Ref*	
Rural	1.19 (0.99, 1.42)	0.057	1.38 (1.13, 1.70)	0.002*
Ethnicity				
Malay^#^	*Ref*		*Ref*	
Chinese	1.00 (0.77, 1.32)	0.976	-	-
Indian	1.08 (0.81, 1.43)	0.593	-	-
Other Bumiputera^+^	1.03 (0.82, 1.29)	0.814	-	-
Others	*Ref*	0.001*	0.39 (0.24, 0.64)	< 0.001*
Marital status				
Married	*Ref*		*Ref*	
Single	0.03 (0.02, 0.05)	< 0.001*	0.04 (0.02, 0.07)	< 0.001*
Widowed/Divorcee	0.55 (0.44, 0.69)	< 0.001*	0.72 (0.56, 0.82)	0.008*
Education level				
No formal education	*Ref*		*Ref*	
Primary	2.63 (1.96, 3.53)	< 0.001*	2.25 (1.63, 3.10)	< 0.001*
Secondary	2.88 (2.15, 3.86)	< 0.001*	2.69 (1.91, 3.79)	< 0.001*
Tertiary	1.97 (1.43)	< 0.001*	2.85 (1.95, 4.17)	< 0.001*
Employment status				
No	*Ref*	0.654	*-*	-
Yes	0.96 (0.82, 1.13)			
Physical activity				
Inactive	*Ref*		*Ref*	
Active	1.85 (1.53, 2.23)	< 0.001*	1.41 (1.12, 1.78)	0.003*

*CI* confidence Interval, *OR* odds ratio, *aOR* adjusted odds ratio, *Ref* reference category,

^#^Includes indigenous people.

^+^Includes Bumiputera Sabah and Sarawak.

*p < 0.05.

Goodness of fit for final model: Hosmer–Lemeshow statistic:0.332.

## Discussion

The aim of this study was to determine the prevalence of receiving a Pap smear test in the past three years, as well as its relationship with socio-demographic factors and physical activity. We discovered that only 35.2% of respondents had undergone a Pap smear test in the past three years. This figure represents just half of the target set by WHO’s Global Strategies for Cervical Cancer Elimination, which aims for 70% of women worldwide to be screened regularly for cervical diseases with a high-performance test [6]. In China and Thailand, the rates of cervical cancer screening were 43.4% and 59.7%, respectively [12, 13]. Among Southeast Asian women, embarrassment, time constraints, and a lack of screening knowledge were identified as barriers to cervical cancer screening [26]. Conversely, age, advice from healthcare workers, and education status, were identified as facilitators of cervical cancer screening among Southeast Asian women [26]. Based on the recognised barriers and facilitators of cervical cancer screening, perhaps more efforts and interventions could be made to enhance the prevalence of cervical cancer screening.

Respondents aged 35–59 were found to have a higher likelihood to undergo a Pap smear test than those aged 18–34. These findings are consistent with what has been reported in the literature from around the world. Young Koreans are more likely to participate in cervical cancer screening as they get older [27]. A systematic review and meta-analysis of Ethiopian women found that women who were in their 30s were 4.58 times more likely to use cervical cancer screening services than those aged 21–29 [15]. In Spain, women aged 25–65 were 5.13 times more likely than those aged 15–24 to undergo a cytology test [14]. Similarly, the prevalence of cervical cancer screening was higher among women aged 35–49 years than women aged 15–24 years among Kenyan women [28]. Growing older may be associated with increased disease risks, resulting in an increase in the frequency of clinic visits and the likelihood of disease screening, including cancer screening. Nonetheless, the likelihood of older persons having cervical cancer screening may decrease due to the American Cancer Society’s 2020 guideline update, which advises against screening for those aged 65 and older, if a series of past tests were normal [29].

According to our study, single and widowed/divorcee respondents were less likely than married respondents to have undergone a Pap smear test. This aligns with a cross sectional study of 403 Jamaican women that found single women were less likely to undergo a Pap smear test [30]. According to a national panel study, being married is associated with higher Pap test rates [31]. In the northeast region of Thailand, being married is a significant predictor of cervical cancer screening adherence [32]. Married women are more likely to access obstetric and family planning services, as well as health care professional promotion and education, and thus may be less hesitant and embarrassed to undergo a Pap smear test.

Our study indicated that respondents from rural localities were more likely to receive a Pap smear test. This might be because rural Malaysians are more likely to undergo a Pap smear test. Cervical screening uptake was 48.9% among rural Malaysians, which was higher than the overall prevalence found in this study [33]. Frequent visits to healthcare facilities can lead to increased testing opportunities. In Malaysia, rural areas have greater fertility rates than urban areas [34, 35]. Higher fertility might increase demand for maternal healthcare services, and therefore more frequent healthcare visits, providing more opportunities for testing.

Additionally, our study found that respondents with higher physical activity levels were more likely to undergo a Pap smear test. This is consistent with prior studies [16, 31]. A study of 1,971 American Indian women found that increasing physical activity was associated with higher likelihood of having a Pap smear test [16]. In a cohort study, women who engaged in vigorous exercise were more likely to receive Pap smear tests [31]. Individuals regularly engage in physical activity may be more willing to participate in health screenings, understanding the benefits of early diagnosis and prevention. Moreover, these may also have a favourable attitude towards health and wellness, which may encourage them to participate in health screenings.

### Strengths and limitations

One of the notable strengths of our study lies in its utilization of a stratified two-stage sampling method, which greatly enhances the representativeness of the sample and ensures that it accurately reflects the demographics of the entire national population. This robust sampling approach contributes to the validity and generalizability of the findings, making them more applicable to the broader Malaysian context. Furthermore, this study holds particular significance as it provides contemporary and nationally representative data on the prevalence of Pap smear screening in Malaysia. By capturing the most up-to-date information, the study offers valuable insights into the current state of Pap smear utilization across the country. These findings serve as a valuable benchmark for evaluating the effectiveness of existing screening programs, identifying areas for improvement, and formulating targeted interventions to enhance cervical cancer prevention and early detection efforts in Malaysia. However, it is also critical to recognise the study’s limitations. Our main concern in this study was the self-reported data collection method for Pap smear tests performed in the past three years, without verification through a medical record review. This could introduce recall bias into the study. Furthermore, this study did not take into account other determinants that could have significant associations with cervical cancer screening.

### Implications and future directions

The Malaysian Ministry of Health recommends Pap smear screening for all sexually active women aged 20 to 65 [36]. If two consecutive annual tests are negative, a repeat screening can be performed every three years [36]. Nonetheless, a substantial non-adherence to the recommended Pap smear screening guidelines was observed (90.5%) among Malaysian women attending health clinics [37]. This finding is alarming, and immediate action is needed to ensure proper adherence to recommended Pap smear screening guidelines to ensure the efficacy of the Pap smear screening programme and policy. Cervical cancer in Malaysia is managed in accordance with the clinical practice guidelines for the management of cervical cancer in Malaysia [38]. A systematic review revealed that women who underwent surgical treatment for cervical intraepithelial neoplasia faced a higher likelihood of experiencing preterm delivery, low birth weight, and preterm premature rupture of membranes before reaching 37 weeks of pregnancy, in comparison to women who did not receive any treatment [39]. Hence, it is essential to conduct further research in Malaysia to investigate the obstetric outcomes of cervical cancer treatment. Such studies are crucial for providing comprehensive care to women, improving reproductive health outcomes, and enhancing their quality of life following cancer treatment.

Given the relatively low rate of Pap smear screening in Malaysia, it is worth considering the implementation of a national program that promotes self-sampling as an alternative approach. Subsequently, it is crucial to conduct comprehensive research to evaluate the effectiveness of this approach in detecting cervical abnormalities and reducing the incidence of cervical cancer. By introducing self-sampling as a part of the national program and conducting rigorous research to assess its efficacy, Malaysia can potentially enhance the accessibility and uptake of cervical cancer screening, leading to improved public health outcomes.

## Conclusions

In conclusion, the prevalence of Pap smear tests conducted in the past three years among respondents was only 35.2%, indicating that overall cervical cancer screening rates remain low. Our study identified several factors associated with higher likelihood of receiving a Pap smear test in the past three years, including being within the age group of 35-59 as compared to aged 18-34, married and being physically active. Measures should be implemented in our country to boost cervical cancer screening coverage and adherence to cervical screening guidelines. Health promotion programmes and policies to enhance awareness about the importance of cervical cancer screening should be strengthened and aimed toward younger women, single women, and widowed/divorcees. The higher cervical screening uptake among rural women should be studied further, and the enabling factors in the rural setup should be emulated in urban areas whenever possible.

## Data Availability

The data supporting the study’s findings are available from the Institute for Public Health, Ministry of Health Malaysia, but access to these data is restricted because they are not publicly available. However, data are available from the author (chanyeemang@moh.gov.my) upon reasonable request and with the permission of the Director General of Health Malaysia.
